# Cell Cycle Stage and DNA Repair Pathway Influence CRISPR/Cas9 Gene Editing Efficiency in Porcine Embryos

**DOI:** 10.3390/life12020171

**Published:** 2022-01-25

**Authors:** Karina Gutierrez, Werner G. Glanzner, Mariana P. de Macedo, Vitor B. Rissi, Naomi Dicks, Rodrigo C. Bohrer, Hernan Baldassarre, Luis B. Agellon, Vilceu Bordignon

**Affiliations:** 1 Department of Animal Science, McGill University, Sainte-Anne-de-Bellevue, QC H9X 3V9, Canada; karina.gutierrez@mail.mcgill.ca (K.G.); werner.glanzner@mcgill.ca (W.G.G.); mariana.priottodemacedo@mail.mcgill.ca (M.P.d.M.); naomi.dicks@mail.mcgill.ca (N.D.); rodrigocbohrer@hotmail.com (R.C.B.); hernan.baldassarre@mcgill.ca (H.B.); 2 Department of Agriculture, Biodiversity and Forests, Federal University of Santa Catarina, Curitibanos 89520-000, Brazil; vbragarissi@gmail.com; 3 School of Human Nutrition, McGill University, Sainte-Anne-de-Bellevue, QC H9X 3V9, Canada

**Keywords:** cell cycle, DNA repair, gene editing, pigs, zygotes

## Abstract

CRISPR/Cas9 technology is a powerful tool used for genome manipulation in different cell types and species. However, as with all new technologies, it still requires improvements. Different factors can affect CRISPR/Cas efficiency in zygotes, which influence the total cost and complexity for creating large-animal models for research. This study evaluated the importance of zygote cell cycle stage between early-injection (within 6 h post activation/fertilization) versus late-injection (14–16 h post activation/fertilization) when the CRISPR/Cas9 components were injected and the inhibition of the homologous recombination (HR) pathway of DNA repair on gene editing, embryo survival and development on embryos produced by fertilization, sperm injection, somatic cell nuclear transfer, and parthenogenetic activation technologies. Injections at the late cell cycle stage decreased embryo survival (measured as the proportion of unlysed embryos) and blastocyst formation (68.2%; 19.3%) compared to early-stage injection (86.3%; 28.8%). However, gene editing was higher in blastocysts from late-(73.8%) vs. early-(63.8%) injected zygotes. Inhibition of the HR repair pathway increased gene editing efficiency by 15.6% in blastocysts from early-injected zygotes without compromising embryo development. Our finding shows that injection at the early cell cycle stage along with HR inhibition improves both zygote viability and gene editing rate in pig blastocysts.

## 1. Introduction

In addition to being one of the main sources of animal proteins for human consumption, pigs have become important animal models for biomedical research because they have characteristics that bear similarities to human features of anatomy, physiology, and metabolism profile [1]. Therefore, their use in research has steadily increased, which has been promoted by advancements in technologies such as transgenesis [2], in vitro embryo production [3], cloning by somatic cell nuclear transfer (SCNT) [4], and more recently, genome editing [5]. All these technologies had significantly contributed to facilitating the manipulation and modification of the pig genome.

The discovery of RNA-guided endonucleases has revolutionized the genome editing process in animals, including pigs. Indeed, the ability of CRISPR/Cas9 to induce double-strand breaks (DSBs) in DNA, guided by single-guide RNAs (sgRNAs) have enabled the creation of changes in genomes in a rapid and precise fashion [6,7]. Gene-edited pigs were first created in 2014 through direct injection of CRISPR/Cas9 components into in vivo-produced zygotes [8], and since then, many studies have applied this technology in swine using zygotes produced either in vivo [9,10,11,12] or in vitro [13,14,15,16].

Although the manipulation of the genome by CRISPR/Cas9-mediated editing is simpler compared to other genome-editing methods (e.g., zinc finger nucleases and transcription activator-like effector nucleases) [17], many factors, such as stage of embryo development, timing of injection, and concentration of injected CRISPR/Cas9 components, can drastically affect embryo viability, editing efficiency, mosaicism rate, and biallelic editing rate [18,19,20,21,22], and thereby degrade the overall efficacy of creating genome-edited pigs. There is evidence suggesting that the injection of CRISPR/Cas9 RNAs into zygotes before the end of the S-phase (DNA synthesis phase) improves gene editing efficiency in embryos [21]. Moreover, it appears that homologous repair (HR) is favored and mosaicism rates are reduced when injections are performed at metaphase II oocytes or at the two-cell stage as compared to zygotes [23,24].

It is known that cells activate DNA damage repair mechanisms after their DNA is cleaved by Cas9. Contrary to somatic cells, which repair damaged DNA mainly by nonhomologous end-joining (NHEJ) pathway [25], embryos seem to preferentially repair their DNA using the HR pathway [26]. Inhibition of the NHEJ pathway, as well RAD51 supplementation or stimulation to improve HR, has been explored as a strategy for improving genome editing (gene knock-in) in somatic cell lines and in zygotes [27,28,29,30].

Here, we evaluated the effect of HR inhibition in combination with the timing of the CRISPR/Cas9 system injection on embryo development and gene editing to create insertion/deletion polymorphisms (indels), in porcine embryos produced in vitro by parthenogenetic activation (PA), in vitro fertilization (IVF), intracytoplasmic sperm injection (ICSI), and somatic cell nuclear transfer (SCNT). Embryos produced by these different technologies were used because they enable evaluating potential effects of chromatin configuration (e.g., two haploid pronuclei in IVF and ICSI, one diploid nucleus in PA and SCNT, undifferentiated bipaternal chromatin in IVF and ICSI, undifferentiated maternal chromatin in PA, and differentiated somatic chromatin in SCNT) on gene editing efficiency.

## 2. Materials and Methods

All the chemicals and reagents used in this study were purchased from Sigma Chemical Company (Sigma-Aldrich; Oakville, ON, Canada), unless otherwise indicated. 

### 2.1. Oocyte In Vitro Maturation (IVM)

Ovaries from prepubertal gilts were collected from a local abattoir (Olymel S.E.C/L.P.) and transported to the laboratory at 32 °C in saline solution (0.9% NaCl) with 100 IU/mL penicillin and 10 mg/mL streptomycin. Cumulus-oocytes complex (COCs) were aspirated from follicles ranging from 3 to 6 mm with a 20 G needle, and only COCs with a minimum of three cumulus cell layers and a homogeneous cytoplasm were selected for in vitro maturation (IVM). Groups of 30 COCs were matured in 90 μL of maturation medium under mineral oil at 38.5 °C with an atmosphere of 5% CO_2_ in humidified air. Maturation medium consisted of TCM199 (Life technologies, Burlington, ON, Canada), supplemented with 20% of porcine follicular fluid, 1 mM dibutyryl cyclic adenosine monophosphate (dbcAMP), 0.1 μg/mL cysteine, 10 ng/mL epidermal growth factor (Life technologies), 0.91 mM sodium pyruvate, 3.05 mM D-glucose, 0.5 μg/mL LH (SIOUX Biochemical Inc., Sioux Center, IA, USA), 0.5 μg/mL FSH (SIOUX Biochemical Inc., Sioux Center, IA, USA), and 20 μg/mL gentamicin (Life technologies, Burlington, ON, Canada). After 22 h of initial maturation, the COCs were washed and transferred to new drops of IVM medium without FSH, LH, and dbcAMP for an additional period of 20–22 h under the same conditions. In vitro matured oocytes had their cumulus cells removed by vortexing in TCM-199 supplemented with 0.1% hyaluronidase. 

### 2.2. Embryo Production and Culture 

#### 2.2.1. Parthenogenetic Activation

Matured oocytes were exposed to ionomycin (15 μM) for 5 min and then transferred to 10 mM strontium chloride in Ca^2+^-free porcine zygote medium (PZM-3) that was supplemented with cytochalasin B (7.5 mg/mL) and cycloheximide (10 mg/mL) for 4 h. Oocytes were then washed and in vitro cultured.

#### 2.2.2. Somatic Cell Nuclear Transfer

Porcine fetal fibroblasts (PFF) were cultured in Dulbecco Modified Eagle Medium/Ham’s F-12 Nutrient Mixture (DMEM-F12), supplemented with 10% FBS (Life Technologies Burlington, ON, Canada) and 1% antibiotics (10,000 U/mL penicillin and 10,000 μg/mL streptomycin) at 37 °C in 5% CO_2_ in air until reaching cell confluency. The matured oocytes were cultured for 1 h in TCM-199 supplemented with 0.4 μg/mL demecolcine and 0.05 M sucrose. This treatment resulted in a small protrusion in the ooplasmic membrane that contained the metaphase chromosomes. The matured oocytes were enucleated in TCM-199 HEPES-buffered medium supplemented with 2 mg/mL of bovine serum albumin (BSA; fatty acid free), 20 μg/mL gentamicin, and 7.5 μg/mL cytochalasin B by removing the chromatin protrusion together with the first polar body. A nuclear donor cell was transferred into the perivitelline space of each enucleated oocyte and then fused electrically using a single DC pulse of 32 V for 70 μs. Electrofusion was performed in a 0.28 M mannitol solution supplemented with 50 μM CaCl_2_, 100 μM MgSO_4_, and 0.1% BSA. To allow cell fusion, the oocytes were transferred to TCM-199 medium supplemented with 3 mg/mL BSA for 1 h. Reconstructed oocytes were then activated as described in the parthenogenetic activation section.

#### 2.2.3. In Vitro Fertilization

Matured oocytes were washed in prestabilized modified Tris-Buffered Medium (mTBM) [31] supplemented with 2 mM caffeine and 0.1% BSA. Fertilization was performed using 2 × 10^5^ sperm/mL of fresh semen in 500 ul of mTBM for 5 h.

#### 2.2.4. Intracytoplasmic Sperm Injection

Fresh semen from fertile boars (supplied by CIPQ Inc.; Roxton Falls, QC, Canada) was washed through centrifugation in TCM-199 HEPES-buffered medium supplemented with 2 mg/mL BSA at 2000 rpm for 2 min. A small fraction of spermatozoa was then transferred to a 10 μL drop of 10% polyvinylpyrrolidone (PVP) in HEPES-buffered TCM-199. Matured oocytes were manipulated in TCM-199 supplemented with 2 mg/mL BSA. A random spermatozoon was immobilized by compressing its tail with a micropipette and a piezo pulse. Using a piezo-micromanipulator (Prime Tech, Tsuchiura, Ibaraki, Japan), the zona pellucida and membrane were perforated, a small volume of ooplasm was aspirated into the micropipette to ensure the membrane was perforated, and then the spermatozoon was injected into the cytoplasm. After sperm injection, oocytes were activated by exposure to 15 μM ionomycin for 5 min followed by 200 μM of Zn^2+^ chelator (TPEN) for 15 min [32].

#### 2.2.5. Embryo Culture

Embryos were cultured in PZM-3 medium under standard conditions [33]. To inhibit the HR pathway, embryos were treated with 10 μmol/L of KU-55933 (an ATM inhibitor; ATMi), which would favor repair by the NHEJ pathway and consequently increase indel rates [26]. The embryos were kept with the inhibitor for 48 h until cleavage assessment. Blastocyst rates were determined at day 7, and embryos that developed to blastocysts were used to determine gene editing rates. 

### 2.3. In Vitro Synthesis of sgRNA

All the primers used in this study were synthesized by IDT (Windsor, ON, Canada). Two sgRNAs were designed using the CRISPR Design software [34] to target each genomic locus (Table S1), and the sgRNAs were produced as described by [35]. In brief, forward primers (59 bp) were synthesized to contain the sequence for the T7 promoter (19 bp) appended to specific sequences targeting each genomic locus (20 bp) followed by sequences (20 bp) from the px330 plasmid (Addgene). The reverse primer (20 bp) was the same for all the sgRNAs, and it targeted the end of the transactivating crRNA sequence in the px330 plasmid (Addgene). 

The PCR products were used as templates for RNA synthesis using the MEGAshortscript kit (Ambion, Burlington, ON, Canada). The newly synthesized RNAs were purified using the MEGAclear kit (Ambion, Burlington, ON, Canada). The quality of synthesized RNAs was visualized by gel electrophoresis. Gene editing experiments involving one locus targeted the X-box binding protein 1 (XBP1; reference sequence: NC_010456.5), or fatty acid binding protein 3 (FABP3; reference sequence: NC_010448.4), or fatty acid binding protein 6 (FABP6; reference sequence: NC_010458.4) genes, and when involving two loci, targeted the FABP3 and FABP6 genes simultaneously. 

### 2.4. Injection of CRISPR/Cas9 System in Zygotes

Injections were performed in TCM199 supplemented with 2 mg/mL of BSA. Cas9 mRNA (20 ng/μL) and sgRNAs (20 ng/μL) were microinjected (10 pL) using a FemtoJet microinjector (Eppendorf). The injections were performed either within 6 h, which corresponds to before/early S-phase (“early” group) or between 14 and 16 h, which corresponds to late/after S-phase (“late” group) post fertilization/activation. After injections, the zygotes were randomly dived into 2 groups and cultured in PZM-3 medium either in the absence (Control) or presence of KU-55933 (ATMi).

### 2.5. Evaluation of Gene Editing

Genomic DNA (gDNA) was extracted from blastocysts as described previously [36]. The purified DNA was used as a template for PCR to amplify the targeted genomic sequences using specific primers (Table S2) and high-fidelity DNA polymerase. The PCR product was analyzed by gel electrophoresis to confirm the size of the amplicon, purified (Monarch DNA Cleanup, New England Biolabs), and then submitted to T7 endonuclease I (T7EI) assay (New England Biolabs). PCR products from individual embryos and from a control (porcine fetal fibroblasts) were mixed together, denatured and allowed to reanneal forming DNA heteroduplexes, as previously described [37,38]. Gene editing was determined by the visualization of the T7EI product on agarose gels, where duplexes composed of mismatched DNA strands showed different mobility patterns compared to homoduplexes (control samples) (Supplementary Materials Figure S1). The T7EI assay results were confirmed in a representative proportion of samples by determining the nucleotide sequences of the edited loci after Sanger Sequencing (McGill University/Génome Québec Innovation Centre).

### 2.6. Statistical Analysis

Statistical analyses were performed by using JMP Software (SAS Institute, Cary, NC, USA). Data were analyzed using ANOVA, and means were compared using Student’s T test and LSMeans Student’s T test for single and multiple comparisons, respectively. Data were tested for normality using the Shapiro–Wilk test. For gene editing analysis, each embryo was considered as an experimental unit, and the T7EI assay results were analyzed as binomial data (i.e., edited or not edited). Differences in edited rates between treatments were analyzed using the chi-squared test. Results are presented as means ± SEM for embryo development or proportions of gene-edited embryos with 95% confidence intervals. *p* < 0.05 was considered statistically significant.

## 3. Results

### 3.1. Time of Injection Affects Embryo Development and Gene Editing Efficiency

**Figure 1:**
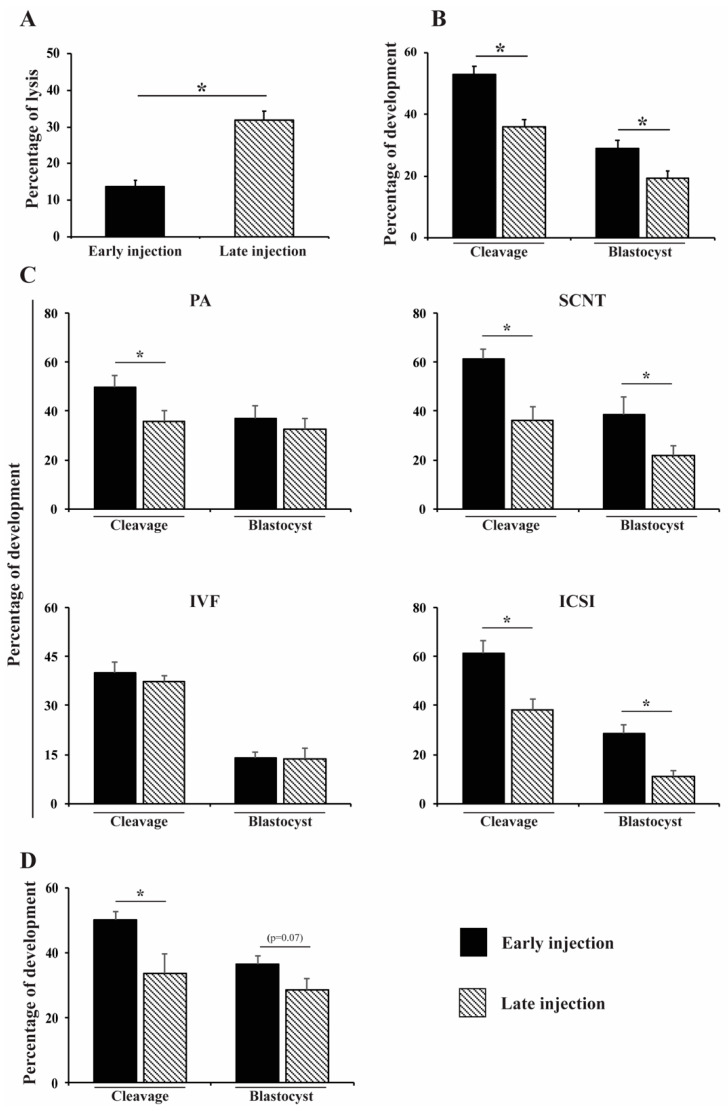
Effect of early and late microinjection during the first cell cycle on embryo development. (**A**) Percentage of membrane lysis after early or late injections. (**B**) Cleavage and blastocyst rates after early or late injections in embryos from all technologies. (**C**) Cleavage and blastocyst rates after early or late injections for each embryo production technology. (**D**) Effect of early or late injections for targeting two gene sequences on cleavage and blastocyst rates of parthenogenetic-activated embryos. Early injections correspond to 0–6 h post activation/fertilization (before/early S-phase). Late injections correspond to 14–16 h post activation/fertilization (late/after S-phase). PA, parthenogenetic activation; SCNT, somatic cell nuclear transfer; IVF, in vitro fertilization; and ICSI, intracytoplasmic sperm injection. Asterisk represents statistical differences (*p* < 0.05). At least six replicates were performed by each embryo production system, containing 30–35 oocytes.

We observed increased membrane lysis and decreased cleavage and blastocyst rates (*p* < 0.05) when gene editing components were injected in late-stage zygotes (Figure 1A,B). Compared to early injection, late injection significantly reduced cleavage rates in PA (13.7% lower), SCNT (25.2% lower), and ICSI (23.1% lower) but not in IVF zygotes (Figure 1C). Late injection significantly reduced development to the blastocyst stage in SCNT (16.7% lower) and ICSI (18.6% lower), but not in PA and IVF embryos compared to early injection (Figure 1C). The same experiment was repeated in PA embryos targeting two different genes simultaneously in the genome. We observed the same trend in cleavage rates when embryos were microinjected at late stages (16.3% reduction–Figure 1D). Thus, regardless of embryo production method, early injection is superior to late injection in preserving embryo integrity and development.

**Figure 2:**
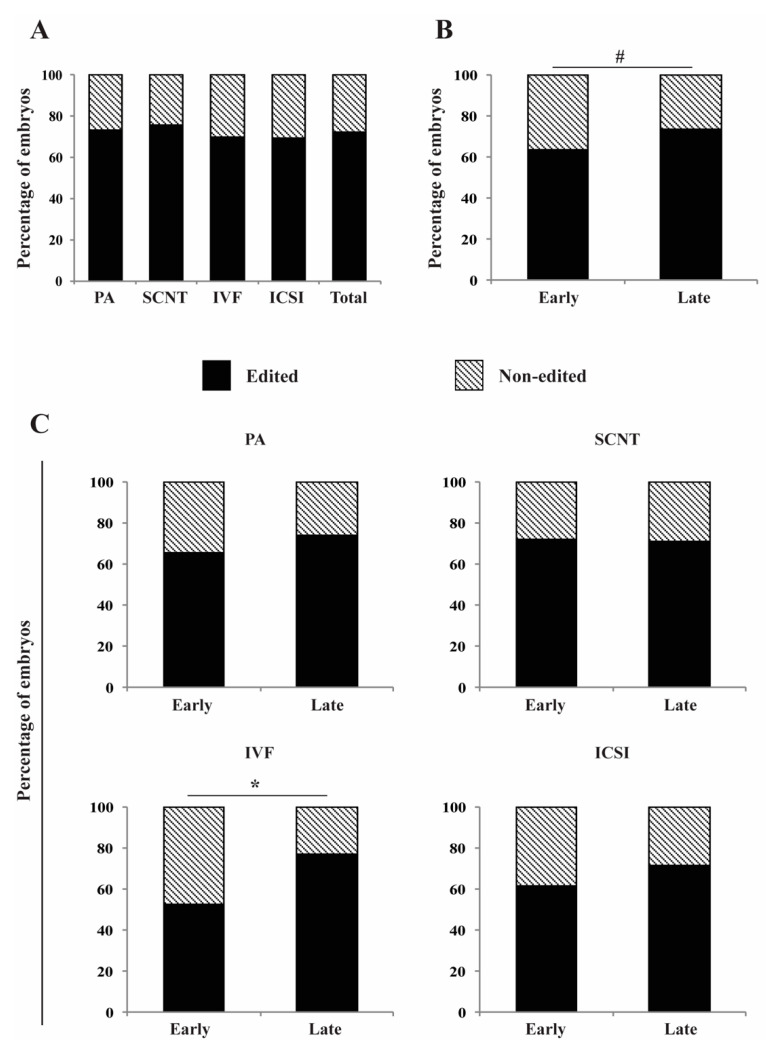
Effect of early and late microinjection during the first cell cycle on gene editing rates. (**A**) Percentage of gene-edited blastocysts from each embryo production technology. (**B**) Percentage of gene-edited blastocysts after early or late microinjections in embryos from all technologies. (**C**) Percentage of gene-edited blastocysts after early or late injections for each embryo production technology. Early injections correspond to 0–6 h post activation/fertilization (before/early S-phase). Late injections correspond to 14–16 h post activation/fertilization (late/after S-phase). PA, parthenogenetic activation; SCNT, somatic cell nuclear transfer; IVF, in vitro fertilization; and ICSI, intracytoplasmic sperm injection. * represents statistical differences (*p* < 0.05) and # represents a tendency (*p* = 0.07). In total, 301 embryos were evaluated (before/early S-phase [*n* = 143]: PA [*n* = 71], SCNT [*n* = 25], IVF [*n* = 21], ICSI [*n* = 26]; after/late S-phase [*n* = 158]: PA [*n* = 94], SCNT [*n* = 24], IVF [*n* = 25], ICSI [*n* = 15]).

The rate of gene editing in blastocysts was 72.3%, and there was no statistically significant difference among the embryo production technologies (Figure 2A). The total rate of gene editing tended to be higher (*p* = 0.07) in blastocysts derived from late-injected zygotes (73.8%) compared to early-injected zygotes (63.8%) (Figure 2B). When averages were compared within each embryo production technology, the gene editing rate in late-injected zygotes was significantly higher (*p* < 0.05) than early-injected zygotes for IVF (77% vs. 52.4%), tended to be higher for PA (74% vs. 65.4%) and ICSI (71.4% vs. 61.5%), and was similar for SCNT (71% vs. 72%) embryos (Figure 2C). Overall, late-stage injection demonstrated improved rates of gene editing.

### 3.2. Inhibition of HR DNA Repair Pathway Improves Gene Editing Efficiency

**Figure 3:**
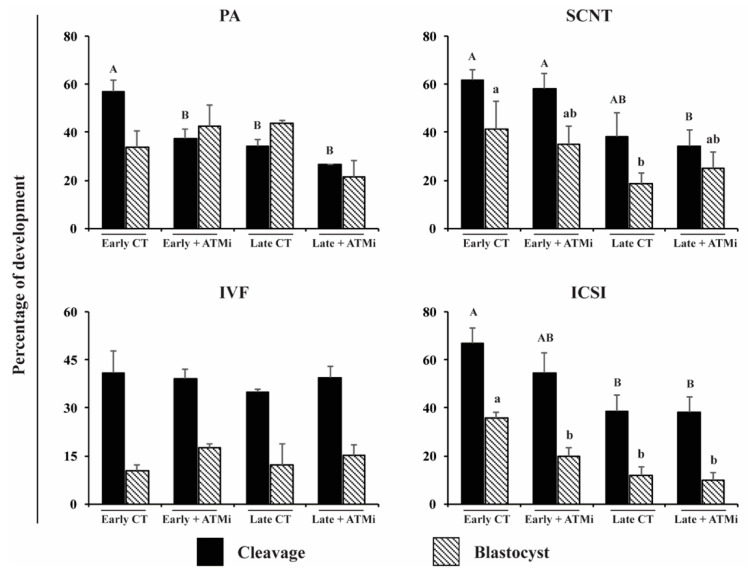
Interaction between time of microinjection and homologous recombination inhibition on embryo development. Cleavage and blastocyst rates after early and late microinjections and treated (ATMi) or not treated (CT) with an inhibitor of homologous recombination in embryos produced by different technologies. Early injections correspond to 0–6 h post activation/fertilization (before/early S-phase). Late injections correspond to 14–16 h post activation/fertilization (late/after S-phase). PA, parthenogenetic activation; SCNT, somatic cell nuclear transfer; IVF, in vitro fertilization; ICSI, intracytoplasmic sperm injection; ATMi, treated with KU-55933 an ATM inhibitor; and homologous recombination pathway). Capital letters indicate statistical differences on cleavage rates, and lowercase letters indicate statistical differences on blastocyst rates (*p* < 0.05). At least six replicates were performed by each embryo production system, containing 30–35 oocytes.

Inhibition of the HR pathway of DNA repair by treatment with KU-55933 during the first 48 h of embryo culture had no apparent detrimental consequences on embryo cleavage and development to the blastocyst stage (Figure 3). As observed in the first experiment, zygotes injected at late cell cycle stage had lower rates of cleavage (37% and 34.7%) compared to early-injected zygotes (52.2% and 48%), for control and KU-55933-treated groups, respectively. Blastocyst rates tended to be lower in late-injected zygotes (20% and 18.7%) compared to early-injected zygotes (30.8% and 26.5%), for control and KU-55933 treated groups, respectively (Figure 3). 

**Figure 4:**
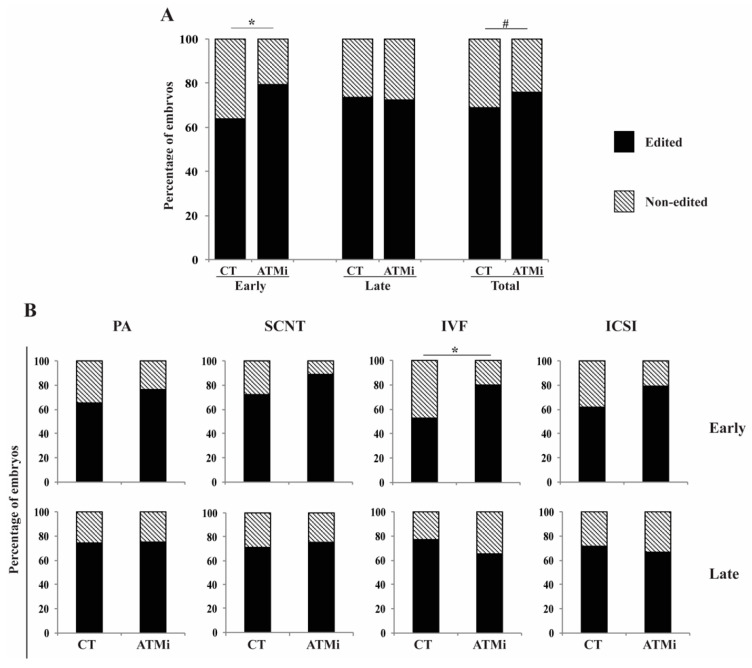
Interaction between time of injection and inhibition of homologous recombination on gene-editing rates. (**A**) Percentage of gene-edited blastocysts after early or late microinjection followed by treatment (ATMi) or not (CT) with an inhibitor of homologous recombination in embryos from all technologies. (**B**) Percentage of gene-edited blastocysts after early or late microinjection followed by treatment (ATMi) or not (CT). Early injections correspond to 0–6 h post activation/fertilization (before/early S-phase). Late injections correspond to 14–16 h post activation/fertilization (late/after S-phase). PA, parthenogenetic activation; SCNT, somatic cell nuclear transfer; IVF, in vitro fertilization; ICSI, intracytoplasmic sperm injection; and ATMi, treated with KU-55933 (an ATM inhibitor; homologous recombination pathway). * represents statistical differences (*p* < 0.05) and # represents a tendency (*p* = 0.07). In total, 571 embryos were evaluated (Early-CT [*n* = 143]: PA [*n* = 71], SCNT [*n* = 25], IVF [*n* = 21], ICSI [*n* = 26]; Early-ATMi [*n* = 139]: PA [*n* = 77], SCNT [*n* = 18], IVF [*n* = 25], ICSI [*n* = 19]; Late-CT [*n* = 158]: PA [*n* = 94], SCNT [*n* = 24], IVF [*n* = 25], ICSI [*n* = 15]; Late-ATMi [*n* = 131]: PA [*n* = 78], SCNT [*n* = 23], IVF [*n* = 20], ICSI [*n* = 10]).

In general, treatment with KU-55933 increased (15.6%) the gene editing rate in blastocysts derived from zygotes injected at the early stages but did not improve the gene editing rate in late-injected zygotes (Figure 4A). Within individual embryo production method, the improvement induced by the treatment was statistically significant in IVF-derived embryos (Figure 4B). 

**Figure 5:**
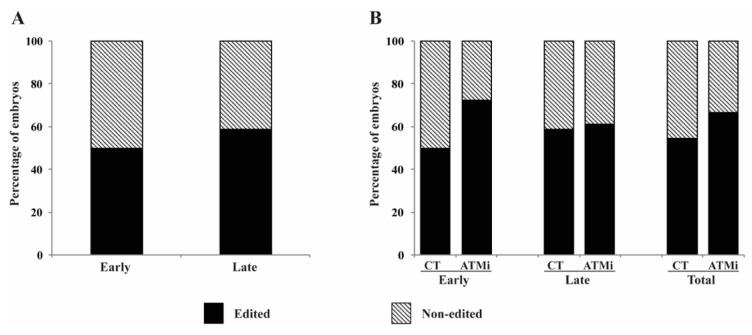
Effect of time of microinjection and homologous recombination inhibition on the rate of double gene editing in embryos produced by parthenogenetic activation. (**A**) Percentage of double gene editing after early or late microinjections. (**B**) Percentage of double gene editing after early or late stages followed by treatment (ATMi) or not (CT) with an ATM inhibitor. ATMi, treated with KU-55933 (an ATM inhibitor; homologous recombination pathway). In total, 72 embryos were analyzed (Early-CT [*n* = 18], Early-ATMi [*n* = 18], Late-CT [*n* = 18], Late-ATMi [*n* = 18]).

The rate of double gene editing was 8.8% higher in blastocysts derived from late-(58.8%) compared to early-(50%) injected zygotes (Figure 5A). Treatment with KU-55933 increased 22.2% (50% untreated vs. 72.2% treated) the rate of double gene editing in blastocyst derived from early-injected zygotes but only 2.3% (58.8% untreated vs. 61.1% treated) in blastocyst derived from late-injected zygotes (Figure 5B).

## 4. Discussion

CRISPR/Cas9 technology has allowed the creation of complex genetic modifications in animals, including pigs [8,9,11,16,39]. However, limitations such as low efficiency, repeatability, efficacy, and high mosaicism rates require improvements to facilitate large-scale applications [9,19,23]. Therefore, the main goal of this study was to determine if zygote cell cycle stage and DNA repair pathway affect the efficacy for producing gene-edited pig embryos derived by different production technologies. 

We first tested if the time when the CRISPR/Cas9 gene editing system was injected into pig zygotes affected embryo viability and gene editing efficiency. Although previous studies have reported that zygote injection decreased embryo survival rates [40,41], it was not determined if stage of the cell cycle at the time of injection is a critical aspect for preserving zygote viability and embryo development. Another study shows no effect on embryo development in zygotes injected 6 h post-IVF [22]. The times of injection (early and late) were selected to correspond to the periods before (early) and after (late) S-phase and were based on previous studies indicating that the S-phase in porcine zygotes starts 5–6 h and ends 15–16 h post activation [42]. We found that late injection decreased zygote viability due to ooplasmic membrane lesion, as well as reduced embryo cleavage and development to the blastocyst stage, suggesting that late S-phase stage zygotes are less resistant to the physical stress induced by micromanipulation and injection. We also found that embryos produced by ICSI and SCNT were more sensitive to injection time than those produced by PA and IVF. This is likely because zygotes derived by ICSI and SCNT undergo more invasive manipulations that include the perforation of the ooplasmic membrane for ICSI and membrane fusion for SCNT. It is also possible that late injection induces damage to the fully developed pronuclei, which are less developed (pre-pronuclei) in early-injected zygotes. Indeed, it is difficult to visualize the location of the pronuclei in porcine zygotes due to their high lipid content [43,44,45]. On the other hand, we observed that zygotes produced by IVF were less affected by injection time. It should be noted that for IVF, it is not possible to determine the exact time of oocyte fertilization. Thus, the stage of the cell cycle at the time of injection was unknown, and the uncertainty may have accounted for the difference observed for IVF embryos. It is also possible that a proportion of the oocytes injected after IVF was not properly fertilized, given the large variations in the fertilization rate and polyspermy that are known to occur in porcine IVF [31,46]. 

In the present study, the analysis of pig embryos that developed to the blastocyst stage revealed higher gene editing rates in those derived from late- compared to early-injected zygotes. A similar trend was observed when comparing rates of double gene editing in the same embryo. This difference may be due to the fact that pig embryos mainly employ the HR pathway to repair DNA double-strand breaks [26]. Hence, a proportion of DNA cleavage induced by Cas9 during the S-phase repaired by HR pathway can escape detection by the T7 endonuclease I assay. Regardless of whether zygotes were injected at early or late S-phase, the injected Cas9 mRNA must first complete translation, which may take several hours to be accomplished, to produce the Cas9 enzyme required for DNA cleavage. Thus, indels may be favored in late-injected zygotes, because gene editing appeared to be favored close to the time when chromatin begins to condense for the first cell division. This timeframe likely does not provide sufficient time for DNA repair via the HR pathway since this process depends on the sister chromatid as a template [47]. When early- and late-injected embryos were compared within each of the in vitro embryo production techniques, we found a statistically significant difference for IVF-derived embryo but not for SCNT-derived embryos. This may be due to SCNT embryos having a single nucleus from the donor cell whereas the IVF embryos initially have two pronuclei derived from the sperm and the oocyte. Pronuclear syngamy (the fusion of male and female pronuclei) occurs at the end of the first cell cycle, which may account for the significant increase in the gene editing rates observed in the late-injected IVF embryos. 

Next, we examined the importance of the DNA repair pathway on the efficiency of gene editing in embryos. Previously, the inhibition of the NHEJ pathway or induction of HR was exploited to increase genome editing (gene knock-in) in somatic cell lines and in zygotes [27,28,29,30]. In these previous studies, their goal was to inhibit the NHEJ pathway or induce HR to improve gene editing in cells and zygotes, whereas our goal was to inhibit the HR pathway with a specific ATM inhibitor to promote repair by NHEJ, which would increase indel events in porcine embryos. We found that inhibition of the HR pathway of DNA repair by treatment with KU-55933 did not decrease embryo development compared to untreated embryos regardless of injection time. This indicates that the modulation of repair pathways during the initial 48 h of embryo development may be a valuable strategy for improving gene editing rates. Furthermore, we observed that KU-55933 treatment significantly increased gene editing rate in early-injected but not in late-injected zygotes. This suggests that DNA repair by the HR pathway significantly contributed to reduced gene editing efficiency observed in early- compared to late-injected embryos. Interestingly, the overall rate of gene editing in early-injected embryos exposed to KU-55933 was higher than late-injected embryos, implying that this strategy can mitigate the developmental issues observed in the late-injected zygotes because it preserves embryo integrity and development, while increasing gene editing efficiency.

The genome editing rates in this study were determined by using the T7EI assay, which is normally applied to detect indels events induced by CRISPR/Cas9 in embryos [19,37,38,48]. Although this is a robust assay, false negative results are possible. For example, T7EI is not efficient at recognizing 1-bp indels [49]. On the other hand, no false positives are detected by the T7EI assay [50]. In this study, we took the additional step of confirming the fidelity of the T7EI assay results by sequencing the PCR product corresponding to the edited region of the genome of some T7EI-positive embryos (Figure S1). Al-though we did not measure the occurrence of mosaicism, the sequencing results suggested it was high, similar to that reported in previous studies [9,23,51]. There is also evidence from previous studies indicating that the occurrence of mosaicism tends to decrease when injections are performed before or at early stages of S-phase compared to the later stages of the first cell cycle [19,21,23,52].

## 5. Conclusions

This study demonstrated that the injection of gene editing components into in vitro-produced zygotes at a later stage of the first cell cycle increased gene editing efficiency, but decreased embryo development compared to injection performed at early cell-cycle stages. Furthermore, the specific inhibition of the HR pathway significantly increased gene editing rates in early-injected zygotes that surpassed the editing rate observed in late-injected zygotes. The porcine species is an important agricultural resource and, more recently, used as models for biomedical research. Improving the efficacy of producing pigs with precise changes in the genome is critical for both animal production and the development of meaningful animal models for research purposes.

## Data Availability

Not applicable.

## Supplementary Materials

The following supporting information can be downloaded at: https://www.mdpi.com/article/10.3390/life12020171/s1, Table S1: Primers used for sgRNA synthesis; Table S2: Primers used for genomic DNA amplification; and Figure S1: Genome editing analysis.
