# Supplementary material for: Cell Cycle Stage and DNA Repair Pathway Influence CRISPR/Cas9 Gene Editing Efficiency in Porcine Embryos
Source: Life (Basel). 2022 Jan 25;12(2):171. doi: 10.3390/life12020171 (PMC8876063; doi:10.3390/life12020171)
Supplement: Supplementary file 1 [file life-12-00171-s001.zip › life-1537354-supplementary.pdf]

life

MDPI

Article

Cell cycle stage and DNA repair pathway influence CRISPR/Cas9 gene editing efficiency in porcine embryos

Karina Gutierrez[1], Werner G. Glanzner[1], Mariana P. de Macedo[1], Vitor B. Rissi[2], Naomi Dicks[1], Rodrigo C. Bohrer[1], Hernan Baldassarre[1], Luis B. Agellon[3,*] and Vilceu Bordignon[1,*]

Supplementary material

Table S1. Primers used for sgRNA synthesis.

Name	Sequence of the forward primers
XBP1_sgRNA1	**(T7)** GCTTGGTGTAGACCATTCGT **(px330)**
XBP1_sgRNA2	**(T7)** CCCCAGCTGATTAGTGTCTA **(px330)**
FABP3_sgRNA1	**(T7)** GCTGGGATTGAGGGACAGGA **(px330)**
FABP3_sgRNA2	**(T7)** GGAAACTCATCCTGGTAAGA **(px330)**
FABP6_sgRNA1	**(T7)** ACTCTCGATCTCATACTTGC **(px330)**
FABP6_sgRNA2	**(T7)** CGATGAGTTCATGAAGCGCT **(px330)**

(T7) corresponds to TAATACGACTCACTATAGG. **(px330)** corresponds to GTTTTAGAGCTAGAAATAGC.The sequence of the reverse primer used for all forward primes is: AAAAGCACCGACTCGGTGCC.

Table S2. Primers used for genomic DNA amplification.

Gene	Sense	Antisense	Amplicon
XBP1	GAGAGCCAAGCTAATGTGGT	TGTCCAGTGACCCTTACCCA	602 bp
FABP3	AGCTGGGCTGTCTGACTCTA	TCCACCCTCCACTATCCCAG	472 bp
FABP6	AATGGGATTCCAGCCAGCAA	TCGCAGCAGTAACATTGGGT	521 bp

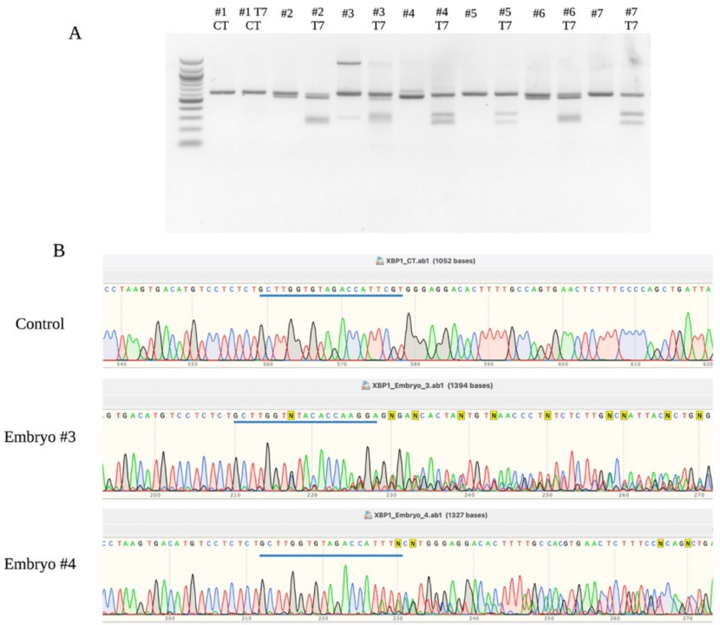

Figure S1. Genome editing analysis. (**A**) Agarose gel resolution before and after T7 Endonuclease I assay. Different pattern of bands is visible in the gel according to the induced mutations. (**B**) Direct Sanger Sequencing from three embryos: one control and two embryos where errors are visible in the chromatogram in the sgRNA position (blue line). This figure was assembled using BioRender.
